# Improving medication availability through mobile wallets and pooled community funds: results from the MoPuleesa hypertension intervention in rural Uganda

**DOI:** 10.1093/heapol/czag044

**Published:** 2026-04-02

**Authors:** Vasanthi Subramonia Pillai, Caterina Favaretti, Andrew Basenero, John Bosco Ntambara, Ivan Wesva, Kafeero Martin Jonathan, Richard Munana, Juliette Cazier, Till Bärnighausen, Josephine Schwab, Jonas Wachinger, Robert Kalyesubula, Shannon A McMahon, Nikkil Sudharsanan

**Affiliations:** Professorship of Behavioral Science for Disease Prevention and Health Care, Technical University of Munich, Am Olympiacampus 11, 80809, Munich, Germany; Munich Center for Health Economics and Policy (M-CHEP), Munich, Germany; Professorship of Behavioral Science for Disease Prevention and Health Care, Technical University of Munich, Am Olympiacampus 11, 80809, Munich, Germany; Munich Center for Health Economics and Policy (M-CHEP), Munich, Germany; African Community Center for Social Sustainability, Kiwembe Zone, Nakaseke Town Council, Nakaseke District, 28993, Uganda; African Community Center for Social Sustainability, Kiwembe Zone, Nakaseke Town Council, Nakaseke District, 28993, Uganda; African Community Center for Social Sustainability, Kiwembe Zone, Nakaseke Town Council, Nakaseke District, 28993, Uganda; African Community Center for Social Sustainability, Kiwembe Zone, Nakaseke Town Council, Nakaseke District, 28993, Uganda; African Community Center for Social Sustainability, Kiwembe Zone, Nakaseke Town Council, Nakaseke District, 28993, Uganda; mTOMADY GmbH, c/o Berlin Institute of Health, Bertolt-Brecht-Platz 3, 10117, Berlin, Germany; Heidelberg Institute of Global Health, Heidelberg University, Im Neuenheimer Feld 672, 69120, Heidelberg, Germany; Heidelberg Institute of Global Health, Heidelberg University, Im Neuenheimer Feld 672, 69120, Heidelberg, Germany; Heidelberg Institute of Global Health, Heidelberg University, Im Neuenheimer Feld 672, 69120, Heidelberg, Germany; African Community Center for Social Sustainability, Kiwembe Zone, Nakaseke Town Council, Nakaseke District, 28993, Uganda; Department of Community Health and Behavioral Sciences, School of Public Health, College of Health Sciences, Makerere University, Mulago Hill Road, Kampala, 7072, Uganda; Heidelberg Institute of Global Health, Heidelberg University, Im Neuenheimer Feld 672, 69120, Heidelberg, Germany; Professorship of Behavioral Science for Disease Prevention and Health Care, Technical University of Munich, Am Olympiacampus 11, 80809, Munich, Germany; Munich Center for Health Economics and Policy (M-CHEP), Munich, Germany; Heidelberg Institute of Global Health, Heidelberg University, Im Neuenheimer Feld 672, 69120, Heidelberg, Germany

**Keywords:** cardiovascular disease, community financing, drug provision, health financing, noncommunicable disease, participatory research, rural health

## Abstract

In Uganda, frequent shortages of antihypertensive medications hinder continuity of care, undermining blood pressure management. Building on preliminary ethnographic research, this study evaluates a community-led, mobile-wallet-based pooling intervention—MoPuleesa—designed to improve medication access at a rural clinic in Nakaseke District, Uganda. Over a 7-month period, 183 patients enrolled and were linked to a digital savings platform that required monthly contributions of 5000 UGX (∼USD 1.39) into a communal fund to bulk-purchase medications at a discounted cost. Using survey data, transaction logs, and clinic records, we assessed contribution behavior, risk of adverse selection, equity, changes in medication availability, and patient blood pressure levels. On average, 48% participants contributed each month. Contribution rates showed no significant differences across education levels or medication costs, suggesting minimal equity concerns or adverse selection. Government pharmacies fulfilled only 8% of total prescriptions; however, for contributors, MoPuleesa closed 84% of the remaining medication gap. However, despite improvements in medication supply, we did not observe statistically significant improvements in blood pressure. Our findings demonstrate the feasibility and effectiveness of mobile money pooling in addressing chronic medication shortages. MoPuleesa achieved broad participation and equitable outcomes in a resource-constrained setting and significantly improved medication availability. We conclude that mobile-based fund pooling for medication can significantly improve medication supply and, with improvements in eligibility assessments, could serve as a complementary or intermediate solution to structural barriers in under-resourced health systems.

Key Messages

- Essential medication shortages are increasingly common in low- and middle-income countries (LMICs) and have a significant impact on health care delivery.
- Community pooling of funds has been used across countries to cover emergencies and unexpected expenses, including in rural Uganda to address gaps in hypertension medication availability. However, because these schemes are informal, paper-based, and lack systematic processes, it remains unclear if all individuals can contribute and benefit, and whether they meaningfully improve medication supply.
- We evaluate a novel mobile money–based savings scheme to facilitate bulk purchase of hypertension medication in a noncommunicable disease clinic in rural Uganda. We find that mobile money–based community pooling significantly improved medication supply, filling the medication gap by nearly 84%, and may represent a promising and adaptable short-term solution for addressing medication gaps in critical chronic conditions.
- Combining community-based financing initiatives with existing national health programs and leveraging active government support in the medication supply management could potentially enhance long-term sustainability.

## Introduction

Public healthcare facilities worldwide are responsible for consistently providing essential medications at no or minimal cost. However, in low- and middle-income countries (LMICs), facilities often face supply disruptions and stockouts. For example, in South Africa, Nemutandani et al. (2020) record over 600 stockout instances of essential medicines across 231 healthcare facilities over 2 years (Nemutandani et al. 2020). Similarly, in an Ethiopian general hospital, Tefera et al. (2022) find that over a 6-month period, the average stockout duration is 39 days (Tefera et al. 2022). These shortages are often far more pronounced in rural and remote geographies. In Uganda, for example, Masters et al. (2014) find that rural health facilities have a 59% higher stockout odds compared with urban facilities (Masters et al. 2014). Such frequent shortages can hinder clinicians’ ability to provide appropriate care and impact patient health outcomes.

The existing literature provides several reasons for stockouts, including insufficient funding for medications (Poku et al. 2017, Anand et al. 2020), suboptimal supply chains (Vledder et al. 2019), inconsistent pricing strategies (Cameron et al. 2009), poor inventory management (Leung et al. 2016), and human resource constraints (Waako et al. 2009). Rural areas face additional logistical challenges in regularly transporting supplies to remote locations (Olutuase et al. 2022, Zuma 2022). This combination of factors makes rural healthcare facilities especially vulnerable to stockouts, and indeed, Master’s et al. (2014) find that the highest share of stockouts is borne by low-level community public health facilities in rural settings (Masters et al. 2014).

Our study evaluates a novel, co-designed solution to address shortages of antihypertensive medications in a rural district of Uganda (Nakaseke). Available medication is typically only a small fraction of what is required, and often, facilities go months without any hypertension medications. Gaps in antihypertensive medication supply have substantial implications for patient health, as an estimated 28.5% of adults in central Uganda (the region that includes Nakaseke) have hypertension; yet, blood pressure (BP) control rates remain alarmingly low (Guwatudde et al. 2015).

To address this challenge, local community members collaborated with the African Center for Social Sustainability (ACCESS)—a nonprofit organization that provides healthcare services in Nakaseke—to develop an informal financial pooling mechanism aimed at reducing stockouts. Individuals requiring antihypertensive medicines contributed funds to a common pool, which ACCESS staff then used to buy medications in bulk from public distribution centers in Kampala, the capital city. This bulk-purchasing strategy was meant to allow patients to obtain medications at significantly lower prices than those charged in local private pharmacies.

Pooling funds to finance health expenditures and economic shocks is common in LMICs (Gugerty 2007, Raccanello and Anand 2009) and has been found to increase savings (Abimbola O et al. 2020) and enhance financial empowerment (Lukwa et al. 2022). As countries move toward universal health coverage, a range of financing approaches have emerged, with co-financing increasingly recognized as a potential pathway to achieving this goal (Collins et al. 2023). These arrangements can be viewed as hybrid financing models, where multiple financial instruments are used to fund health service delivery. Hybrid financing arrangements are particularly common in LMIC settings where public service delivery is weak or underfunded (Onabowale 2020, Machete and Marques 2021, Razali 2026).

While the existing program in Nakaseke demonstrated strong community willingness to self-finance medications, it also raised operational and economic concerns. Operationally, relying on physical cash deposits increased risks associated with securely storing funds, limited scale-up potential, and increased adminstrative complexities in tracking which patients were eligible to receive medications. Economically, there were four key challenges. First, it was unclear how many patients would be able and willing to contribute the estimated amount (5000 Ugandan Shillings, ∼USD 1.39) required to maintain the bulk-purchasing model. Second, it was uncertain whether the program equally benefited all population groups or disproportionately benefited wealthier households with a greater ability to contribute. Third, achieving a cost-effective and balanced fund would require both individuals with modest and more extensive medication needs to contribute to the pool. If only individuals with the greatest need contributed, this would increase the financial strain on the pool and require much higher overall contributions to be sustainable [an issue known as adverse selection (Akerlof 1978)]. Finally, and most importantly, despite the introduction of the paper-based system, there was no evidence on the extent to which this pooling mechanism improved medication supply in real-world settings and translated into improved BP control.

To address these operational challenges, evaluate key economic issues, and measure the program’s effect on medication supply and BP, we partnered with ACCESS and mTOMADY (a nonprofit mHealth organization) and co-designed a mobile-wallet-based version of the community pooling system with key stakeholders and intervention end-users—referred to as “MoPuleesa” (Mo for Mobile, Puleesa, the Lugandan term for BP). MoPuleesa is an example of a hybrid financing intervention that uses digital technologies to facilitate coordination, transparency, and timely resource mobilization. Our intervention seeks to provide an adaptive, intermediate solution to improve health access through community pooling and to address short-term needs and priorities while longer-term public financing or institutional capacity is simultaneously being strengthened. We implemented MoPuleesa for 7 months with 183 patients receiving hypertension care in a local public primary care clinic in Nakaseke. Our results aim to both evaluate MoPuleesa and inform future efforts to leverage mobile money and pooled funds to address medication shortages in Uganda and similar settings.

## Methods

### Study setting

This study was conducted in Semuto, a sub-county in the rural Nakaseke district of central Uganda with an estimated population of 191 100 (Nakaseke District Local Government 2018). Most residents rely on small-scale farming as their primary source of income, earning an average of 71 229 Ugandan Shillings per month (approximately USD 20) (Mbolanyi et al. 2016). Hypertension (or high BP)—a leading cause of several cardiovascular conditions (Prospective Studies Collaboration 2002, Fuchs and Whelton 2020)—is a significant health concern in this area, affecting an estimated 28.5% of the population in Uganda's central region, which includes Nakaseke (Guwatudde et al. 2015 ).

We conducted the study at the noncommunicable disease (NCD) treatment unit in Semuto Health Center IV, one of the district’s public primary healthcare facilities. The clinic, managed by ACCESS Uganda, operates once a week on Wednesdays and serves ∼250 patients diagnosed with hypertension. During each clinic session, patients with hypertension receive brief health education, have their BP assessed, consult with a medical officer, and—when supplies permit—obtain a 1-month supply of antihypertensive medications at no cost in line with government mandates. They are encouraged to return monthly for BP monitoring and medication refills.

### Intervention development

Our study drew on a participatory, human-centered design (HCD) approach to co-develop the intervention and facilitate feasibility, acceptability, and success. Formative research as part of the first HCD phases highlighted an existing, informal pooled-financing initiative for procuring hypertension medication in the study setting. This initiative was accepted and appreciated by the community members, but the administrative challenges of running an informal paper-based scheme impeded broader success. Over the course of the subsequent co-design process, a multidisciplinary team conducted iterative in-depth interviews and focus group discussions to engage patients, community members, community advisory boards, and clinical staff, gathering feedback on intervention strategy (including envisioned contribution amounts and medication distribution pathways). Further details about the larger HCD process is published as a separate paper (Schwab et al. 2023).

### MoPuleesa mobile platform

#### Platform design and registration

MoPuleesa is a mobile-based payment platform that was developed by mTOMADY, and co-designed with ACCESS Uganda, local community members, and other key stakeholders to address the specific needs and constraints of the local population. MoPuleesa enables participants to transfer money from their personal mobile money wallets into a communal fund dedicated to purchasing antihypertensive medications in bulk. The platform functions as a digital ledger and accounting tool, offering a transparent method for recording and managing patient contributions. For further details on the co-design process, please refer to our published protocol (Schwab et al. 2023); a separate paper describing lessons learnt from the co-design activities has been accepted for publication elsewhere and is forthcoming.

In our study, we registered all interested and eligible patients at the Semuto Health Center IV on MoPuleesa and linked the platform to their existing personal mobile wallet at the time of recruitment. Mobile wallets have achieved widespread adoption throughout the region; however, if a person did not have their own mobile wallet, they had the opportunity to connect MoPuleesa to a close family member’s account after obtaining permission from that family member. Once registered, users could access the platform through Unstructured Supplementary Service Data (USSD) menus on their mobile phones. These menus allowed the users to check their account balances, contribute funds, and review transaction histories. Unlike internet-dependent applications that require smartphones, USSD operates over standard mobile networks and does not need internet access, making it particularly suitable for rural settings where smartphone ownership and mobile data coverage may be limited or unreliable.

All contributions were automatically recorded and consolidated into a pooled account managed by a coordinator at ACCESS Uganda, with support from mTOMADY. This coordinator could view detailed contribution records, including the contributors’ IDs, transaction amounts, and whether each transaction was successful.

#### Contribution and medication disbursement

Typically, patients collected medications monthly during their regular clinic visits, provided the medications were available. To receive medications purchased through the pooled fund, participants were required to contribute 5000 Ugandan Shillings (approximately USD 1.39) via the MoPuleesa platform by the last Wednesday of the preceding month (e.g. to receive medications at any of the February 2024 clinic days, participants had to have contributed by the last Wednesday of January). This amount was determined through consultations with community members and ACCESS staff to strike a balance between generating sufficient funds for bulk purchasing and minimizing the financial burden on households, while ensuring that a large share of patients can realistically contribute to maximize the total pool of funds. Based on current market prices for hypertension medications at private pharmacies around Nakaseke and our participants’ medication needs, we estimate that patients would spend an average of 18 988 UGX (5 USD) per month on their prescriptions (excluding secondary costs such as additional travel, missed work, due to one or more required separate trips to pharmacies). Therefore, the contribution of 5000 UGX is highly cost-saving for patients. The last Wednesday of the month was chosen as the deadline, to align with clinic schedules (facilitating in-person reminders and sufficient time for ACCESS staff to procure medication before the next clinic day).

#### Medication purchasing

Each month, an ACCESS staff member traveled to Kampala to purchase antihypertensive medications using the pooled community fund and transported them back to Nakaseke. Upon arrival, the medications were stored separately from government-provided supplies in a designated, locked space under the supervision of ACCESS personnel.

### Participant recruitment and study timeline

#### Target population and eligibility

We recruited participants from the weekly hypertension clinic at Semuto Health Center IV. Participants were eligible if they (1) had a prior diagnosis of hypertension or presented with uncontrolled BP at the current visit (verified through clinic records of that day) and (2) had access to a mobile phone with a mobile wallet, either their own or that of a family member willing to contribute on their behalf.

While the exact number of patients receiving hypertension care at Semuto Health Center IV is not precisely documented, ACCESS estimates the clinic to serve ∼250 individuals. Our study included the majority of the clinic’s hypertension patient population.

### Patient journey and study procedures

#### Recruitment visit

**Figure 1:**
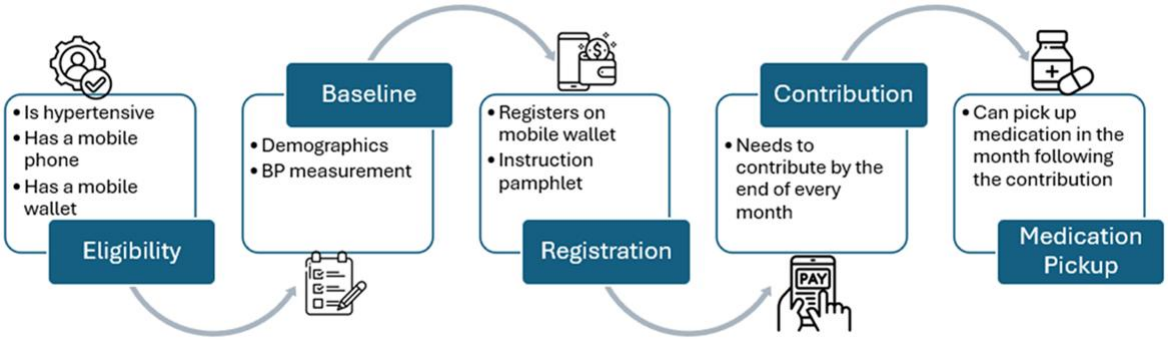
Patient journey, Nakaseke, Uganda, 2023. The icons in the image are drawn from Flaticon.com.

Figure 1 illustrates the patient journey. On recruitment days, patients arrived at the clinic and waited for their consultation with the medical officer. During this waiting period, a clinic staff member measured each patient’s BP, recorded the reading in both the patient’s paper-based medical notebook and the electronic system, and assigned them a unique ID. On recruitment days (all Wednesdays in August and September 2023), we approached potential participants in the clinic’s waiting area after their BP measurement but before they consulted with the clinical officer. All interested patients were then directed to an ACCESS data collector, who provided information about the study, obtained informed consent, assessed eligibility, and, for those who consented and qualified, collected basic demographic information. All data collection was conducted digitally using mobile phones via RedCap.

Regardless of their participation in the study, all patients then waited in a common area before their consultation with the medical officer. After their consultation, eligible participants met with another ACCESS staff member who introduced the MoPuleesa initiative and invited them to register. Registration was free, and participants retained the option to deregister or refrain from using the service at any time. The ACCESS staff member guided registrants through the enrollment process, explained the payment schedule and medication disbursement system, and provided a demonstration of how to navigate the USSD menus and make payments. Participants were explicitly informed that making a contribution in a given month guaranteed them access to medications in the following month, whereas noncontributors would continue to rely on the government supply, subject to availability. To ensure participants fully comprehended and remembered the procedure, we also provided them with a pamphlet with detailed instructions and key deadlines for contributions.

#### Follow-up visits: medication disbursement and verification of payment

Enrolled and contributing participants were eligible to receive medications from the pooled fund supply starting from the first monthly visit following recruitment. On each clinic day, all individuals consulted with a clinical officer, who prescribed medication as required. A facility staff member asked enrolled participants to first visit the government medication supply and receive as much of their prescribed medications as possible from the available government stock, which operates on a quasi-first-come, first-served basis due to limited stock. As the number of patients and total medication needs are not known at the start of each clinic day, staff attempt to ration medications equitably; however, patients who arrive earlier often receive a larger portion of their prescriptions.

After receiving any available government-supplied medications, participants proceeded to an ACCESS Uganda staff member responsible for the pooled-financed supply. Using a web-based interface, the staff member verified whether participants had made the minimum contribution (i.e. 5000 UGX) by the monthly deadline. Patients who had contributed received any remaining medications required to fulfill their prescription. Individuals who did not contribute did not qualify for the pooled fund supply and thus had to rely exclusively on the government stock.

#### Timeline

**Figure 2:**
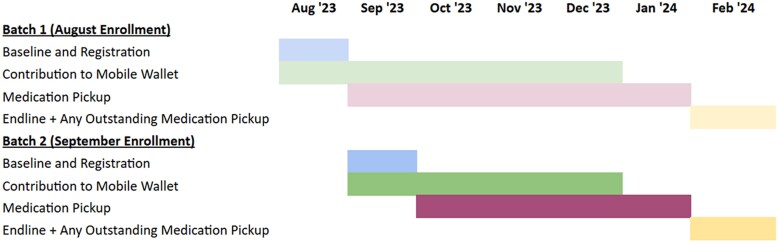
Study timeline, Nakaseke, Uganda, 2023.

The study began on 2 August 2023 and continued until 29 February 2024. We had two batches of recruitment: one in August and one in September. Participants were eligible to contribute each month from the time of enrollment until December 2023. Since participants received their medications in the month following their contributions, the final scheduled disbursements were made in January 2024. We conducted the end line survey and completed any outstanding disbursements in February 2024. Figure 2 describes the timeline in detail. We pre-registered our study with the German Clinical Trial Registry (DRKS00030922).

#### Data sources

We draw on five main data sources for this study:

1. Baseline demographics: During recruitment, we collected basic demographic information, including age, sex, and educational status, through a baseline questionnaire.
2. Contribution records: We used MoPuleesa ledger metadata to track contributions made to the pooled fund. This included details on the date of transfer, the amount transferred, and whether the transaction was successful.
3. Medication needs: From the first month after recruitment, surveyors recorded the details of each participant’s prescription at the time of medication disbursement, providing information on their monthly medication requirements throughout the study period. We documented the types of medications prescribed, prescribed quantities, prescription durations, and medication prices from pharmacies around Nakaseke.
4. Medication disbursement records: Throughout the study period, staff maintained digitized records indicating the type and quantity of medications received by participants and their source (government supply or pooled fund).
5. BP records: BP was measured at three key points: at baseline, during each patient visit throughout the study period, and at end line. Trained staff measured and maintained digital records of these BP values.

### Outcomes

We focus on two primary outcomes: (1) contributions to the pooled fund and (2) medications received by the participants. We focus on BP measured as a secondary outcome, as our primary aim was to improve medication supply rather than tackle other barriers, such as adherence, that mediate medicine availability to BP improvements.

We first examine participants’ capacity and willingness to contribute to the pooled fund by focusing on two measures. First, we use a binary indicator denoting whether a participant contributed in a given month. Second, we examine each individual’s contribution rate, calculated as the proportion of months in which the participant successfully contributed relative to the total possible months of contribution. Since participants enrolled at different times (August or September), the maximum possible number of monthly contributions differed accordingly (up to five for those who enrolled in August and up to four for those who enrolled in September).

We measure coverage of medication supply by comparing each participant’s prescribed medications, converted into a total pill count, against the actual number of pills they received per visit. This calculation shows the extent to which each participant’s prescription was fulfilled either by the government supply or by the pooled fund.

### Statistical analyses

We begin by presenting the baseline demographic characteristics of the participants in our sample and then investigate several aspects of the MoPuleesa program.

First, we investigate participants’ willingness and ability to contribute to the mobile money scheme by reporting the share of successful transactions and the average proportion of months in which they made contributions. We also evaluate fixed thresholds of participation, such as the proportion of individuals who contributed every eligible month, 5 greater than or equal to 50% of eligible months, less than 50% of eligible months, and those who never contributed. These thresholds are based on a binary measure that indicates whether participants contributed during each eligible month.

Second, to explore equity considerations, we replicate this analysis while stratifying participants by socioeconomic status, measured through their highest completed level of schooling (categorized into 4 groups: no formal schooling, less than primary school, primary school completed, and secondary education or higher).

Third, we assess the potential for adverse selection by examining whether the contribution pool is disproportionately composed of individuals with higher expected medication costs, which reflect their medication needs and the prices of the medications if purchased from a private pharmacy. During data collection, we recorded the different types of medications prescribed and the required daily pill intake for each participant. Clinicians prescribed from a selection of six different antihypertensive medications, tailoring their choices based on each patient's condition. Based on participants’ initial prescriptions (before receiving any study-provided medication) and corresponding prices from local private pharmacies, we estimated total monthly medication costs for each participant. On average, patients received prescriptions for 2 types of medication during their initial visit. At prevailing private pharmacy prices, these initial prescriptions correspond to an average monthly cost of 18 988 UGX. We stratified these monthly medication costs into tertiles (less than 8400 UGX for the low tertile, 8401–19 920 UGX for the middle tertile, and 19 921–81 440 UGX for the high tertile). We then analyzed the contribution rates across these tertiles.

Fourth, we estimate the extent to which the program enhanced medication supply by calculating the proportion of total prescribed pills dispensed from both the government supply and the mobile money–funded supply. We stratify these results by whether participants contributed to the fund (and were thereby eligible for the supplemental supply). As individuals receive medications from the government supply before accessing the pooled fund, the remaining portion of the prescription not covered by the government serves as a measure of the medication supply gap. We also assess the extent of spillovers, where patients who did not contribute to the pooled fund received medications from the mobile money–funded supply.

Finally, while the intervention primarily focused on improving access to medication, a secondary aim was to assess whether this improvement in medication access led to downstream improvements in BP control. To examine whether better medication access improved BP levels, we measure the association between picking up medications and participants’ mean systolic BP in the subsequent month. We focus on the systolic values because systolic hypertension is the most common type of high BP among adults in Uganda (Shakil et al. 2022). Importantly, a large body of evidence shows that systolic BP is more strongly and consistently associated with cardiovascular disease incidence and mortality (Psaty et al. 2001, Borghi et al. 2003). Since isolated diastolic hypertension is relatively uncommon (Shakil et al. 2022), medical guidelines prioritize systolic BP thresholds, making it a more clinically and policy-relevant target for hypertension interventions. To analyze this, we ran a linear regression with the participants’ mean systolic BP as the dependent variable, whether the participant picked up medication in the previous month as the main independent variable, and baseline BP, sex, and age of the patient as control variables. This measures whether and to what extent picking up medications is associated with participants’ BP levels. Additionally, we examined whether patients who picked up medication more often had better BP control. To evaluate this, we ran a linear regression with the participants’ end line systolic BP as the dependent variable, the total number of times the participant picked up medication through the study as the main independent variable, and the baseline BP measurement, sex, and age of the participant as control variables.

## Results

### Sample description and demographic characteristics

The study began in August 2023 and concluded in February 2024. In total, we surveyed 230 patients from the NCD Semuto clinic, ∼90% of the total patient pool. After excluding individuals who declined to participate (*N* = 28), did not have hypertension (*N* = 21), lacked access to a mobile wallet (*N* = 2), or were unable to complete online registration (*N* = 19), our final sample comprised 183 participants. Of these, 80% were recruited in August 2023 and 20% in September 2023.

Table 1 summarizes the sample’s baseline characteristics. Participants were, on average, 61 years old (SD = 12.8), and 82% (*N* = 146) were female. Fifteen percent of participants had no formal education. At baseline, participants’ mean systolic BP was 142 mmHg (SD = 21.5), and their mean diastolic BP was 81 mmHg (SD = 12.38). Overall, 54% of the sample had uncontrolled BP at enrollment.

**Table 1:** Baseline demographic characteristics, Nakaseke, Uganda, 2023.

Characteristics	Total sample (*N* = 183)
Age (in years)	61 (12.77)
Sex	
Female	146 (82%)
Male	32 (18%)
Education	
No formal schooling	22 (15%)
Less than primary school	58 (38%)
Primary school completed	45 (30%)
Secondary education or higher	26 (17%)
Blood pressure	
Systolic	142 (21.50)
Diastolic	81 (12.38)
Participants with uncontrolled BP	96 (54%)

Mean (SD); *N* (%); Uncontrolled hypertension is defined as systolic BP ≥ 140 mmHg or diastolic BP ≥ 90 mmHg. Data availability varied across variables: sex and age were available for 178 participants, baseline BP for 177 participants, and education for 151 participants

### Contribution rates

**Figure 3:**
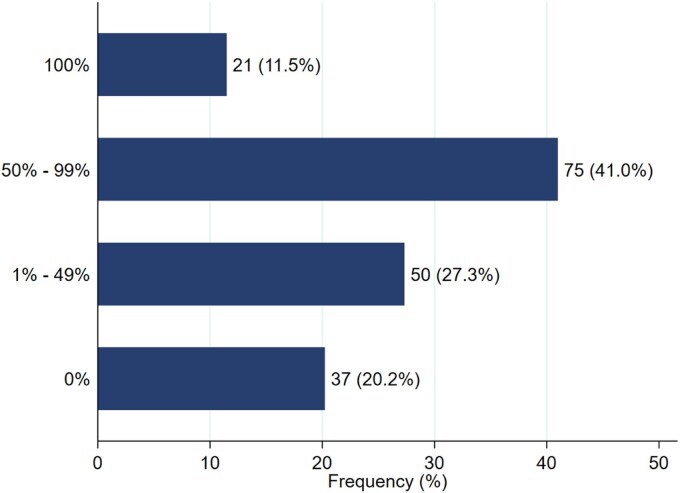
Contribution rates (*N* = 183), Uganda, 2023.

We recorded 495 transactions, of which 86% were successful, 12% failed due to incorrect inputs (such as entering the wrong PIN) or other technical challenges, and 2% failed due to insufficient funds in the mobile money wallet. We observed a moderately high willingness to contribute to the pooled medication fund. Overall, participants contributed 48% of the eligible months. In any given month, an average of 47% of participants successfully contributed to the pooled fund. Categorically, 11.5% of participants contributed every eligible month, 41% contributed more than half of the eligible months (50%–99%), and 27.3% contributed less than half of the eligible months (1%–49%). Only 20.2% of participants never contributed (Fig. 3).

### Equity considerations

**Figure 4:**
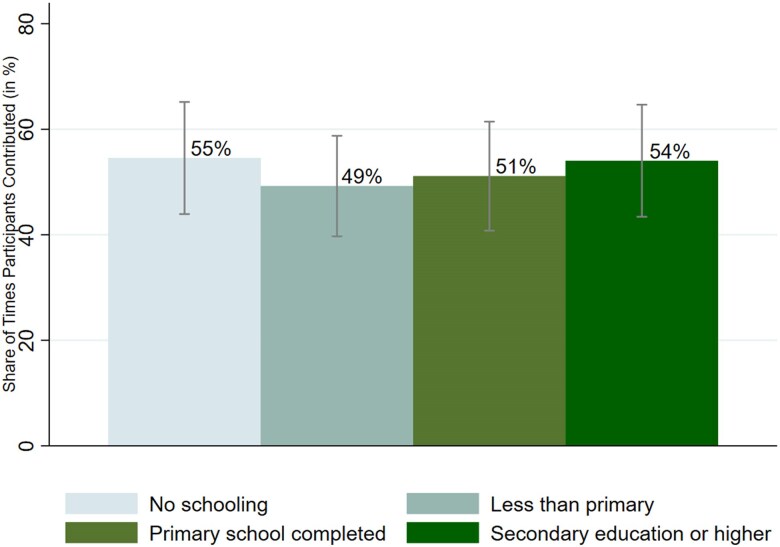
Equity: contribution rates by education (*N* = 151), Uganda, 2023. Error bars represent 95% confidence intervals.

While overall contribution levels were promising, an important consideration was whether participation was skewed toward wealthier and more advantaged households. In Fig. 4, we present contribution rates stratified by level of education, testing for differences across socioeconomic groups.

We did not find evidence of differential contribution across socioeconomic groups. The mean contributed share was 55% for those with no formal education, 49% for those with less than primary education, 51% for those who completed primary education, and 54% for those with secondary education or more. A pairwise mean test indicated no statistically significant differences between the groups (*P* > 0.5 for all between-group tests).

### Adverse selection

**Figure 5:**
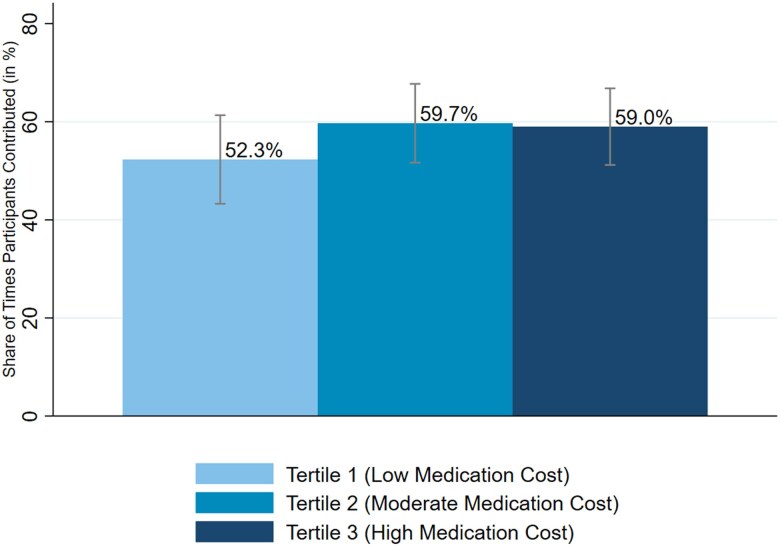
Contribution rate by participants’ medication costs (*N* = 151 participants), Uganda, 2023. The above analysis is restricted to those who attended at least one follow-up consultation during the study period, as prescriptions were measured only when participants attended their follow-up consultation and not during enrollment. Low medication costs: <8400 UGX pills, Moderate medication costs: 8401–19 920 UGX, High medication costs: 19 921–81 440 UGX for the high tertile. Error bars represent 95% confidence intervals.

Comparing contributions based on medication costs did not provide strong evidence of adverse selection (Fig. 5). Contribution rates among individuals in the middle and highest tertiles were nearly identical (59.7% vs 59%). Although individuals in the lowest-cost tertile contribute marginally less often (52.3% vs 59.7%) than those in higher-cost tertiles, this difference was not statistically significant (*P* = 0.24). This suggests that individuals with the higher cost burdens were not disproportionately selected into the pooled fund.

### Medication delivery

We evaluate the impact of MoPuleesa on medication delivery by comparing the proportion of individuals’ total prescriptions dispensed through the government supply, the pooled fund, or left undispensed. This analysis is stratified based on whether an individual contributed to the pooled fund.

Since individuals first interacted with government supply staff, these staff members did not know whether a participant had contributed to the pooled fund. As a result, they should not have strategically dispensed medications—for instance, by providing fewer medications to contributors under the assumption that they would receive additional supplies from the pooled fund. Therefore, all individuals should have received medication based solely on the government supply’s availability and the dispensers’ assessment of demand (see Section Follow-up Visit Medication Disbursement and Verification of Payment for a detailed description of the prescription process). Any gap in the total prescription after receiving the government supply reflects the extent of medication shortages in the existing stock. While this approach highlights the extent of the gap, an important related question is how much of the shortfall could be covered by the pooled fund.

**Figure 6:**
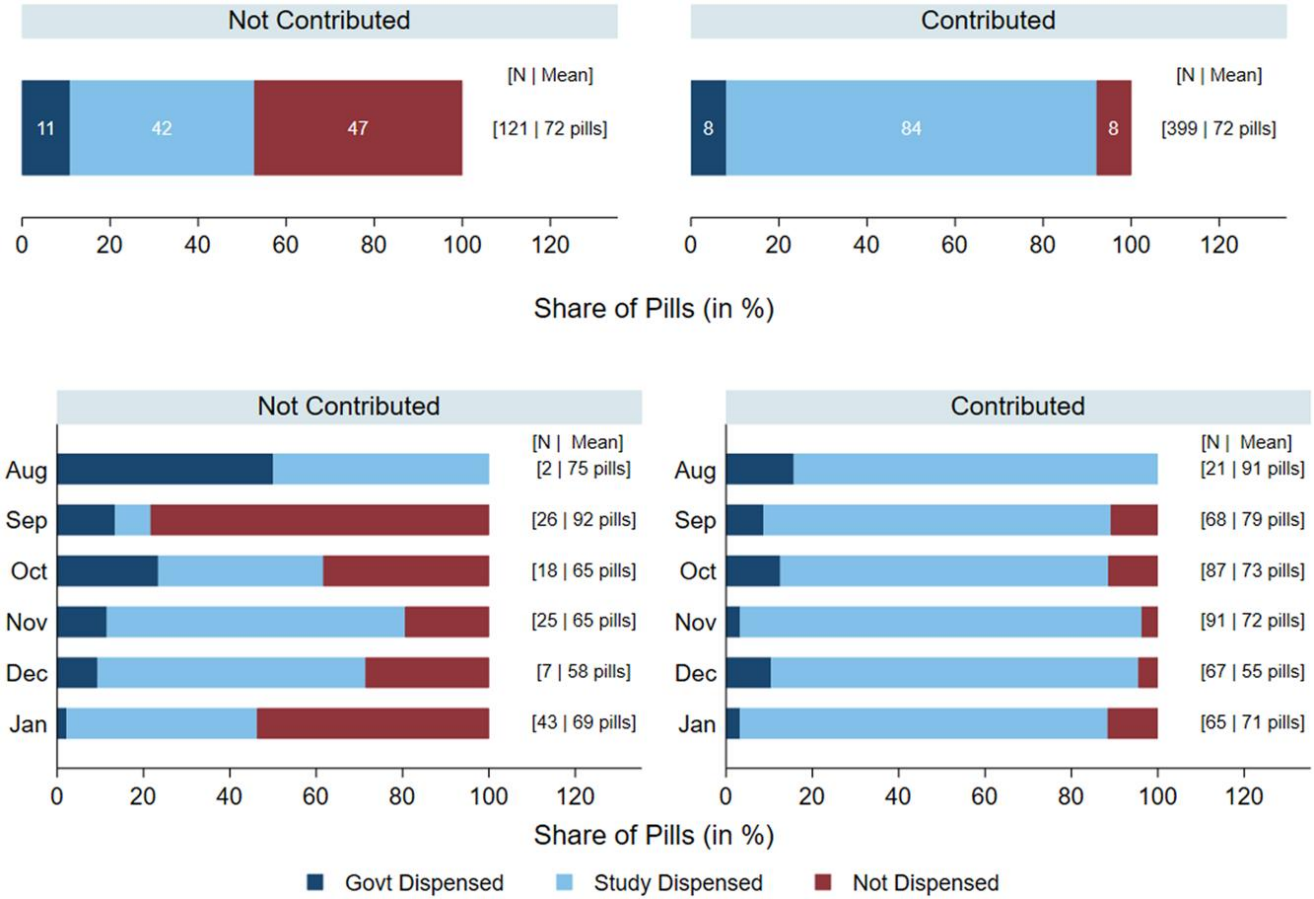
Pills dispensed per patient per visit by contribution Status (*N* = 520 visits) Uganda, 2023. The figure above shows the share of pills dispensed by the government-funded dispensary, study-funded dispensary, and the share left undispensed. “N” indicates the total number of patient visits during a given month, and “Mean” indicates the mean pills prescribed per patient per visit. The above analysis is restricted to those who were prescribed any medications at their follow-up visit (*N* = 151 individuals).

Figure 6 compares the average number of prescribed pills dispensed per month between contributors and noncontributors. There are large supply gaps in the government supply, which, on average, covered only 8% of total prescription needs. However, the pooled fund effectively addressed most of the prescription shortfall. Among individuals who contributed, the pooled fund covered 84% of the remaining 92% prescription gap, leaving only 8% of the total prescriptions unfulfilled. This remainder was due to difficulties in accurately forecasting monthly needs and instances where doctors prescribed medicines that were not available through wholesale channels. These results remained consistent when analyzed across months, indicating no significant variation over time.

Additionally, diverging from original intervention protocols, we find that some individuals who had not contributed still received medications from the pooled fund. This resulted in 42% of the prescription gap for non-contributors being covered by the pooled funds. While the process for determining whether an individual had contributed and was thus eligible for pooled fund medications was assumed to be straightforward, staff reported difficulties in consistently verifying participants’ contribution status despite attempts to create a transparent and accessible verification interface. This was primarily because some patients collected their medications in months other than their scheduled follow-up visits, which complicated tracking and required additional efforts to reconcile outstanding disbursements.

### BP control

We do not find evidence that participants who picked up their medications in the previous month had an improved BP level during their subsequent visit compared to those who did not pick-up medications (Table 2). The average systolic BP of those who picked up medications was 1.99 mmHg lower than those who did not. However, this result is not statistically significant (*P* = 0.3). We also do not find evidence of an association between the frequency of medication pick-up and BP (Table 3).

**Table 2:** Monthly medication pick-up and mean systolic BP, Uganda, 2023.

	Systolic BP (in mmHg)
Picked up medication in the previous month (reference group: those who did not pick-up)	−1.99 (1.97)
Sex (female)	−0.48 (2.66)
Age	0.12 (0.11)
Baseline systolic BP	0.39*** (0.05)
Ref. group mean	140.05
R-squared	0.17
Total observations	379

**Table 3:** Frequency of medication pick-up and mean systolic BP, Uganda, 2023.

Frequency of medication pick-up	−0.62 (0.93)
Sex (female)	6.86* (3.56)
Age	0.31** (0.14)
Baseline systolic BP	0.32*** (0.09)
R-squared	0.15
Total observations	155

In A, we present the mean systolic BP for those who picked up medication in the previous month compared to those who did not pick-up. In B, we present the association between the number of times participants picked up the medication and their end line BP. Standard errors are displayed in parentheses. *P < 0.1, **P < 0.05, ***P < 0.01.

## Discussion

We developed and implemented a mobile money–based pooling system (MoPuleesa) to purchase hypertension medications for patients at an NCD clinic in Nakaseke, Uganda. Over our study period, government pharmacy stocks remained consistently low, with large medication delivery gaps. However, MoPuleesa was highly successful at addressing these shortages, by closing 84% of the medication supply gap.

A key finding is that approximately half of the participants contributed every eligible month. Several factors may explain gaps in contribution. First, transaction metadata suggests that 12% of attempts failed due to technical challenges or incorrect inputs (e.g. entering the wrong PIN). These patterns suggest that while addressing technical barriers could meaningfully enhance the effectiveness of future initiatives, it is unlikely to close the full contribution gap. Second, although participants received a pamphlet in the local language detailing procedures and deadlines during enrollment, forgetfulness, or poor comprehension of monthly payment deadlines may have led to missed contributions. Finally, although minimum contribution amounts were decided via consultations with key stakeholders to reflect feasibility and preferences, financial constraints and income irregularities among the study population may have impeded regular contributions. Our ongoing qualitative work aims to better understand participants’ ability to engage in the scheme and their responses to potential future increases in contributions.

An important concern with pooling funds is the potential for adverse selection, where patients with a higher medication cost burden would contribute more frequently, leading to higher overall contribution rates for all participants. However, we did not find evidence of adverse selection, with nearly equal contribution rates among participants with the highest and lowest medication costs. Another key challenge of patient self-financing programs, is whether they result in an inequitable distribution of resources, whereby individuals with greater means contribute more often and thus benefit more. However, we did not find any evidence of differences in contribution rates across patients from different levels of socioeconomic status. These findings alleviate key economic concerns and suggest that MoPuleesa and strategies leveraging mobile-based self-financing can be viable for large population groups in rural LMIC settings like Nakaseke.

Beyond improving medication supply, an extended aim of this project was to assess whether better access to medications improved BP levels. Our results show that the mean systolic BP did not improve for those who received medications compared to those who did not. Importantly, this null result was not driven by low power: based on a post-hoc statistical power analysis, we were powered (at 80%) to detect a difference of 5.7 mmHg between participants who picked up medications and those who did not. A 5 mmHg reduction in systolic BP is often considered a clinically meaningful effect in hypertension care trials (Schwalm et al. 2019, Rahimi et al. 2021). Our sample size was thus powered to determine whether the intervention had a meaningful effect on systolic BP. These results were based on associations and should not be interpreted as causal evidence. Several factors may have contributed to the lack of differences in systolic BP between those who did and did not receive medications through the program. Although we tracked how often participants collected their medications, we did not capture actual adherence, which is essential for BP control. The null effects on BP could also reflect suboptimal treatment receipt, as even those who contributed received, on average 84% of their necessary medications. Beyond pharmacological treatment, lifestyle factors such as poor diet and lack of physical activity may have hampered efforts to improve BP. Taken together, these results suggest that while improved medication supply is a necessary condition and increases the opportunity for better hypertension management, patient adherence to treatment and lifestyle changes remain critical factors in achieving BP control.

Another important takeaway from implementing this digital money pooling system is the incidence of spillovers between contributors and noncontributors. We found that 42% of noncontributors received medications, while 8% of contributors did not. This mismatch was primarily driven by a gap in the mobile application’s design, since the platform did not directly indicate eligible participants. Instead, enumerators accessed backend metadata, downloaded lists of eligible participants, and manually verified eligibility during medication disbursement. Designing future iterations of the digital platform to automatically indicate participant eligibility based on contributions and medication pick-up could substantially reduce such errors. These spillovers may also partly reflect community tensions arising from unequal access. This may include patients demanding medications despite not contributing, or perceived moral pressure on program staff to dispense drugs even to those who are not eligible. We believe that co-designing the program with participants, alongside transparent implementation processes, can help preempt this risk by embedding fair and collectively agreed-upon mechanisms for medication eligibility.

From a health systems perspective, scaling such interventions requires broader structural changes. In many Sub-Saharan African and LMIC settings, medication stockouts are often driven by supply chain inefficiencies in transportation, inventory management, and demand forecasting (Wagenaar et al. 2014, Leung et al. 2016). Such issues could be partly addressed by integrating additional digital components into interventions like MoPuleesa to help pharmacies generate more accurate demand estimates and enhance supply planning, especially with the Ministry of Health actively promoting health records digitization across Uganda. A broader question that this study raises is whether it is appropriate to rely on self-financing to meet individuals’ health care needs. The public healthcare sector in LMICs is often heavily resource-constrained, resulting in low insurance coverage, shortages in human resources, and significant gaps in treatment availability. In response to these challenges, community-driven approaches, such as self-financed peer support groups (Sanya et al. 2023), community health clubs (Waterkeyn and Cairncross 2005), and successful self-financing systems such as rotating savings and credit associations, are a common feature in several LMICs (Gugerty 2007, Abimbola O et al. 2020, Lukwa et al. 2022).

Moreover, MoPuleesa is a hybrid financing mechanism that serves as a transitional strategy, bridging short-term needs and longer-term reforms. By addressing financing gaps and sharing risk across stakeholders, the hybrid financing model improves the feasibility of service provision in contexts where no single source of finance is sufficient on its own. Such hybrid mechanisms could also advance universal health coverage by encouraging intersectoral collaboration between health systems, private providers, and community organizations to address systemic barriers to healthcare provision (Obi et al. 2024, Otchere et al. 2026). While the long-term goal of health policy should be structural improvement of healthcare delivery systems, such innovative, intermediate community-driven solutions—if aligned with local priorities and with community ownership—offer useful pathways for managing healthcare needs and improving patient outcomes in the short run.

It is important to note that this study was implemented based on an existing community-led program and in partnership with a trusted local NGO, within a community familiar with pooled-financing arrangements and with high mobile money penetration. While these context-specific factors likely contributed to effective implementation and may limit generalizability, we believe that several aspects of the underlying design can be leveraged and adapted in other LMIC contexts. First, much of Sub-Saharan Africa has experienced rapid growth in mobile money use for everyday transactions (World Bank Group 2022). Second, countries across Sub-Saharan Africa and other LMICs have long-standing experience with pooled-financing mechanisms such as informal savings and lending groups, and mutual aid arrangements (Oraro and Wyss 2018, Lukwa et al. 2022). Finally, community health workers played an integral role in implementing this study and, given their widespread presence across LMICs, could effectively support similar initiatives. As such, the key transferability challenge is not introducing the concept of mobile money or securing community buy-in on fund pooling but rather formalizing these mechanisms specifically for health financing to improve reliability. Additionally, efforts to strengthen local NGO capacity to facilitate, operationalize, and manage such arrangements would be crucial for future studies, as their involvement is key to building community trust, improving efficiency, and ensuring successful implementation.

Our study also found that the pooled fund was insufficient to cover all costs, and discussions with local stakeholders indicated that an increase in contributions to 10 000 UGX would be necessary to maintain the program. This revised rate may impede participants’ willingness and ability to pay in the long run. However, one potentially effective strategy to enhance MoPuleesa's sustainability would be to design this co-financing mechanism as a complement to existing government health financing programs, rather than the sole source of medication financing. This blended financing approach could improve program affordability and strengthen long-term viability. Additionally, structuring MoPuleesa as a supplementary tool may increase government buy-in and facilitate better alignment with national health financing strategies.

We contribute to a growing body of work highlighting the positive impact of mobile money technologies in the healthcare sector. The widespread adoption of mobile wallets has been shown to enhance both healthcare access and delivery. Ahmed and Cowan (2021) find that mobile money transfer technology increased usage of health services across 1800 households in East Africa by improving the potential for informal borrowing as well as easing payment mechanisms for medical expenditures (Ahmed and Cowan 2021). Similarly, Egami and Matsumoto (2020) find that mobile money systems increased antenatal care take-up by improving liquidity for geographically remote households (Egami and Matsumoto 2020). Hamani et al. (2023) also find that mobile money improved payment turnaround times and reduced cash leakages for community health worker incentives in Senegal (Hamani et al. 2023). However, the existing literature on evaluating digital money for pooling mechanisms, especially for healthcare financing, is scarce. Some studies have examined digital payments and savings groups more broadly, though not specifically in the context of health. For example, Francois and Squires (2021) find that using mobile money networks to run ROSCAs in the Democratic Republic of the Congo substantially increases contributions to the pool (Francois and Squires 2021). Similarly, Mehmood et al. (2019) find that digital money has the potential to strengthen saving groups by mitigating issues in record-keeping, payment collection, and distribution (Mehmood et al. 2019). Therefore, our study adds an important extension to the work on mobile money penetration by assessing whether it helps streamline and improve informal savings and pooling systems for healthcare access in resource-limited settings.

Our study has important limitations. First, 54% of our participants had uncontrolled BP, and women comprised 82% of the sample. This contrasts with national survey data, where hypertension prevalence is more evenly distributed by sex and 70%–90% of diagnosed individuals have uncontrolled BP (Musinguzi and Nuwaha 2013, Uganda Hypertension Profile 2023, Mondo et al. 2025). This difference is likely because our study was conducted among patients attending a dedicated rural NCD clinic rather than among the general population. Indeed, literature from Uganda and across Sub-Saharan Africa generally finds higher levels of health system engagement among women than men (Yeatman et al. 2018, Sikka et al. 2021). Our results are thus best interpreted as being relevant for and generalizable to those who are already connected with the health system. Second, this intervention was limited to hypertension and did not include other chronic diseases. However, this allowed us to focus on one of the leading risk factors for cardiovascular mortality with exceptionally high burden in the study population, respond to community-driven priorities, and rigorously assess the feasibility of the digital intervention. Finally, our study was conducted over 6 months and does not assess long-term impact. However, this duration was considered appropriate (Pladevall et al. 2010, Webster et al. 2018) to assess intervention feasibility as BP has been found to respond to treatment within hours and fully take effect within weeks of treatment initiation (Salam et al. 2024).

We report on an innovative strategy to address stockouts of antihypertensive medications in rural public healthcare centers in Nakaseke, Uganda. By introducing a mobile money savings scheme, we fill 84% of the medication gap for patients and improve access to hypertension care. While the magnitude of this improvement inevitably varies with local supply chain constraints, our findings demonstrate that the program generated notable end-user uptake and effectively addressed supply shortages in Nakaseke.

## Data Availability

Data will be made available upon request.

## Reflexivity statement

Our research was conducted by a diverse, multinational team with expertise spanning behavioral and development economics and global health. The project was conceived jointly, with authors from all settings involved in study design, data collection, and analysis. Local co-authors provided critical contextual insights that shaped the study implementation. We held regular team discussions to examine how our assumptions, perspectives, and interactions could shape the research question and interpretation of our findings. Through these practices, we ensured transparency, ethical rigor, and collaborative knowledge creation while acknowledging the influence of our collective standpoint on the research process.
